# Post-traumatic stress disorder and professional quality of life among psychiatric staff

**DOI:** 10.1192/j.eurpsy.2021.1202

**Published:** 2021-08-13

**Authors:** K. Yaich, S. Omri, N. Smaoui, R. Feki, M. Maalej Bouali, J. Ben Thabet, L. Zouari, N. Charfi, M. Maalej

**Affiliations:** Psychiatry C Department, Hedi chaker University hospital, sfax, Tunisia

**Keywords:** post-traumatic stress disorder, professional, psychiatric staff, quality of life

## Abstract

**Introduction:**

Psychiatric staff could be exposed to various types of violence that might have potential consequences on their psychological balance.

**Objectives:**

To detect post-traumatic stress disorder (PTSD). To assess the professional quality of life among psychiatric hospital workers.

**Methods:**

A descriptive cross-sectional study was conducted in the psychiatric department of the Hedi Chaker University Hospital in Sfax. The questionnaire study had three major components: the baseline participant characteristics,the post-traumatic stress disorder Checklist (PCL-5) for which a total symptom severity score cutoff of 38 was recommended as the cutoff for a positive screening test and the Professional Quality of Life Scale (ProQOL).

**Results:**

Thirty-one participants completed the questionnaire. The sex-ratio was 0.93. The mean age was 41.5 years. All participants were exposed to physical or verbal assault. Physical aggression was the most traumatic behavior reported by 39.3% of psychiatric professionals. A feeling of insecurity when performing professional tasks was reported by 93.3% of participants. Among participants, 41.9% expressed the desire to change workplace. The mean score on the PCL-5 was 21.6 ± 15.35. Five participants (16.7%) had a PCL-5 score ≥ 38. The Compassion Satisfaction mean score was 37.48 ± 5.64. The burnout mean score was 26.41 ± 7.3 and the mean score at the secondary traumatic stress scale was 27 ±6.7.

**Conclusions:**

PTSD could result from stressful events encountered in the course of managing patients in mental health departments. Attention to post-traumatic event interventions may be useful both to reduce the rate of PTSD and to improve the professional quality of life among psychiatric staff.

